# Establishing a Standardized Surveillance System for Health Care-Associated Infections in Vietnam

**DOI:** 10.9745/GHSP-D-21-00284

**Published:** 2022-06-29

**Authors:** Daniella Coker, Ha Thi Kim Phuong, Lan Thi Phong Nguyen, Tran Ninh, Neil Gupta, Tran Thi Thu Ha, Nguyen Tuan Truong, Hoang Van Thanh, Amber Vasquez, Hien Thi Thu Bui, Paul Malpiedi

**Affiliations:** a Centers for Disease Control and Prevention, Atlanta, GA, USA.; b Oak Ridge Institute for Science and Education, Oak Ridge, TN, USA.; c Vietnam Administration for Medical Services, Hanoi, Vietnam.; d Centers for Disease Control and Prevention, Hanoi, Vietnam.; e PATH, Hanoi, Vietnam.

## Abstract

Standardized surveillance for health care-associated infections (HAI) is critical to understand HAI burden and inform prevention strategies at a national level. Due to differing and generally limited resources in Vietnam's health care facilities, implementation of HAI surveillance has been variable and data quality has not been systematically assessed. In 2016, the Vietnam Administration for Medical Services (VAMS) under the Ministry of Health, with the support of partners, began to establish a context-appropriate, standardized HAI surveillance system for bloodstream infections (BSI) and urinary tract infections (UTI) among 6 pilot hospitals in Vietnam. We identified 5 key elements of our HAI surveillance implementation process that have been conducive to ensuring data quality and program sustainability and scalability. These include: (1) engaging stakeholders, (2) designating roles and responsibilities, (3) developing context-sensitive, standardized surveillance protocols, (4) creating a surveillance implementation strategy, and (5) linking HAI surveillance and prevention activities. With the active participation of infection prevention and control staff from the 6 pilot hospitals, standardized HAI surveillance for BSIs and UTIs was expanded to 12 additional hospitals in 2019. Together, VAMS and partners are helping Vietnam fulfill its commitment to safe health care for all patients.

## Abstract (precis)

Standardized surveillance for health care-associated infections (HAI) is critical for HAI prevention, yet standardized implementation across low- and middle-income countries is limited. With the support of partners, the Vietnam Ministry of Health implemented standardized HAI surveillance in 6 hospitals, and in doing so, identified 5 key elements for program success.

## Teaser

**Key Findings:**

- Maintaining stakeholder investment in establishing the foundation for Vietnam's national health care-associated infections (HAI) surveillance system will be key for its growth and longevity.
- Developing HAI surveillance protocols that could be applied consistently across surveillance hospitals based on the local context to ensure the quality of data generated by the surveillance system was critical to the surveillance system's success.
- Focusing initial implementation of standardized HAI surveillance in select intensive care units of 6 hospitals allowed challenges to be addressed and corrected and ensured the long-term sustainability of surveillance in the hospital.

**Key Implications:**

- National authorities should consider engaging stakeholders, designating roles and responsibilities, developing context-sensitive, standardized surveillance protocols, creating a surveillance implementation strategy, and linking HAI surveillance and prevention activities when developing HAI surveillance systems in low- and middle-income countries to increase the likelihood of successful surveillance implementation.

## INTRODUCTION

Health care-associated infections (HAIs) are serious infections acquired while receiving medical treatment in a health care facility. HAIs are a major threat to patient safety, often resulting in prolonged hospital stays; high costs for patients, families, and health care facilities; and preventable deaths.^1^ Limited data on the global burden of HAIs indicate low- and middle-income countries (LMICs) are disproportionally impacted by HAIs (15.5 per 100 patients) compared to high-income countries (7.1 and 4.5 per 100 patients in Europe and the United States, respectively).^2^

Surveillance is defined as the “ongoing systematic collection, analysis, and interpretation of health data essential to the planning, implementation, and evaluation of public health practice.”^3^ Surveillance for HAIs can be used to describe the burden of HAIs, identify high-risk populations and procedures, and evaluate the impact of targeted infection prevention and control (IPC) interventions.^4^ HAI surveillance is a core component of both facility and national IPC programs worldwide.^4^ Yet, in LMICs, many facility surveillance systems use nonstandardized case definitions or suboptimal surveillance methods^2^ and national surveillance systems are often nonexistent.^4^ Continuous HAI surveillance is a resource-intensive process, made more difficult in LMICs with limited human resources, microbiology laboratory capacity, and technical experience designing and implementing such systems.^5^

An effective HAI surveillance system must be standardized; procedures for identifying potential cases, applying case definitions, and reporting outcome measures must be performed uniformly and consistently. Failure to do so can result in the inability to interpret fluctuations of HAI rates as true changes in incidence or as artifacts of applying surveillance procedures inconsistently. Standardization is critical for aggregating HAI data for reporting and may become increasingly challenging as surveillance networks grow.

Lack of standardization in an HAI surveillance system can result in the inability to interpret fluctuations of HAI rates as true changes in incidence or as artifacts of applying surveillance procedures inconsistently.

There are no internationally agreed upon standardized HAI case definitions for implementation across LMICs. The U.S. Centers for Disease Control and Prevention (CDC) and the European Centre for Disease Prevention and Control (ECDC) each offer standardized HAI surveillance definitions, but they both rely upon a degree of capacity (laboratory, diagnostic, and epidemiological) that may not be achievable in certain low-resource settings. Therefore, it is important for LMICs to develop standardized HAI surveillance methodologies that consider the context and available resources of their settings rather than those used in high-income countries.

## HEALTH CARE-ASSOCIATED INFECTION SURVEILLANCE IN VIETNAM

Estimates of the HAI burden in Vietnam are limited by the scarcity of published data from the region.^6^^–^^8^ In 2009, the Vietnam Ministry of Health (MOH) prioritized the national monitoring and prevention of HAIs by issuing an IPC guideline^9^ that included the implementation of HAI surveillance in public and private health care facilities. Due to the differing and generally limited resources in Vietnam's health care facilities, implementation of the guideline has been variable and data quality was often unknown. Some large hospitals in Vietnam were using U.S. CDC definitions across all inpatient care units, which is a time-intensive process that reduces IPC staff's available time for prevention activities. Other hospitals relied on clinical judgment for confirming HAIs, which led to interobserver variation that may have inappropriately affected HAI rates.

**FIGURE 1:**
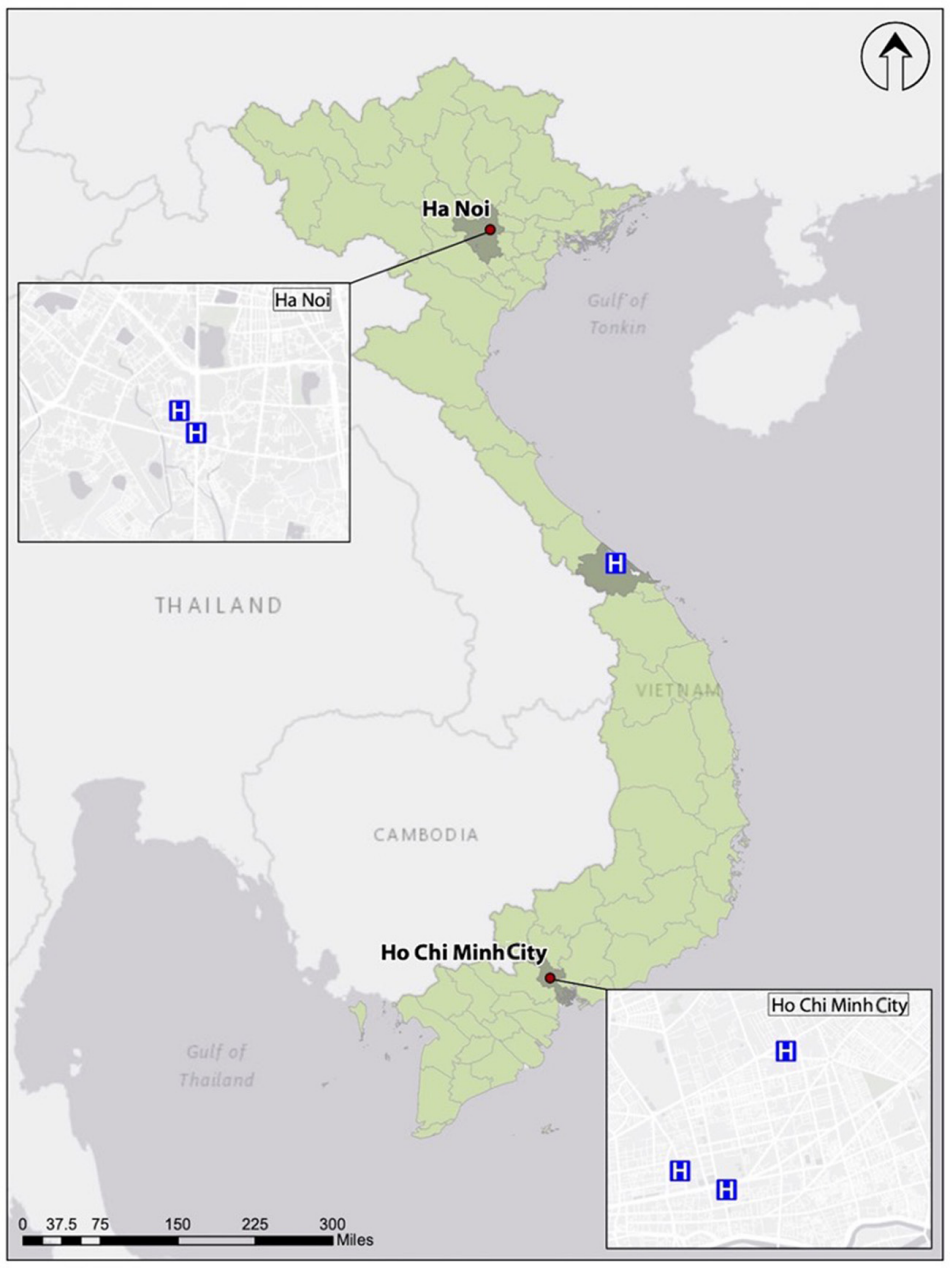
Map of the 6 Pilot Hospitals Implementing Standardized Surveillance for Bloodstream Infections and Urinary Tract Infections in Vietnam

Recognizing the need to develop a simple and resource-appropriate national standardized surveillance system, in 2016, the Vietnam Administration for Medical Services (VAMS) under the MOH, CDC, and PATH collaborated to establish a standardized HAI surveillance system for bloodstream infections (BSIs) and urinary tract infections (UTIs) among select intensive care units (ICUs) at 6 hospitals (Figure 1). We aim to present the experience of surveillance implementation in Vietnam, highlighting elements conducive to sustainability and scalability.

### Ethics Approval

This work underwent CDC review for ethical approval and was determined to not qualify as human subjects research.

## IMPLEMENTATION OF STANDARDIZED HEALTH CARE-ASSOCIATED INFECTION SURVEILLANCE IN VIETNAM

We identified 5 key elements conducive to ensuring data quality and program sustainability and scalability: engaging stakeholders, designating roles and responsibilities, developing context-sensitive, standardized surveillance protocols, creating a surveillance implementation strategy, and linking HAI surveillance and prevention activities.

### Engaging Stakeholders

A critical first step in Vietnam was to establish strong and frequent collaboration between local and international stakeholders. Although commitment and oversight were established at the national governmental level, there was still a need to augment technical capacity by engaging international partners and local IPC experts. VAMS identified international organizations and hospital-based technical experts with IPC experience in Vietnam and abroad and invited them to support the development of the HAI surveillance system.

A critical first step was to establish strong and frequent collaboration between local and international stakeholders.

The health care system in Vietnam primarily consists of public facilities (94%), though there is a growing private sector.^10^ The country's facilities are organized into 4 administrative levels: central (Level I) and provincial (Level II) are the largest and often most well-resourced hospitals, which service populations of up to 2 million; district (Level III) hospitals, which service populations between 100,000 and 200,000; and commune (Level IV) hospitals, which service populations of around 5,000 to 10,000.^10^

IPC program leaders from 6 hospitals (5 central and 1 provincial hospital), who had previously worked closely with VAMS to develop national IPC guidelines and support facility-level IPC implementation, served as local technical experts in the group developing the surveillance system and piloted BSI and UTI surveillance in selected ICUs in their hospitals. To ensure that the HAI surveillance system was developed in a way that complemented existing IPC capacity in Vietnam and did not duplicate efforts, we engaged these key local experts and worked alongside them to adapt U.S. CDC's National Healthcare Safety Network (NHSN) BSI and UTI definitions, develop standardized surveillance protocols, and create and deliver training to hospital staff engaging with the surveillance system. We cultivated stakeholder investment for HAI surveillance by providing on-site technical assistance during periodic hospital visits, which established a regular opportunity for hospital IPC teams to meet with VAMS and international partners. We also sought to solidify administrative support and collaboration through a hospital and MOH delegation visit to the U.S. CDC to continue the conversation of national commitment to HAI surveillance and prevention.

### Designating Roles and Responsibilities

In Vietnam, designating roles and responsibilities between stakeholders was important for building positive working relationships and supporting efficient surveillance implementation. A national antimicrobial resistance unit, consisting of an epidemiologist, physician, information technology officer, and planning officer, was established within VAMS to provide national-level coordination and technical oversight of HAI surveillance data management, analysis, and dissemination in collaboration with VAMS IPC leadership. The antimicrobial resistance unit and VAMS IPC leadership were trained on the technical details of the HAI surveillance system and participated in all training sessions for the hospitals.

Hospital IPC teams were responsible for the implementation and oversight of the surveillance system at their own facilities, which included establishing clear roles and responsibilities for relevant teams within their own hospitals to ensure that protocols were followed and errors were minimized.

CDC and PATH provided technical support to VAMS and hospital surveillance teams throughout implementation, including assisting with protocol development; advising and training hospital surveillance teams on effective implementation; and creating an electronic data reporting, analysis, and visualization system. While CDC and PATH continue to serve as advisors for the HAI surveillance system, VAMS and IPC leaders at the 6 pilot hospitals are now well established as the technical leads for the surveillance system as it expands.

### Developing Context-Sensitive, Standardized Surveillance Protocols

The feasibility of implementing surveillance consistently across participating hospitals was a major factor in deciding which infections to include in Vietnam's HAI surveillance system. Because microbiology laboratories at all 6 pilot hospitals were able to perform bacterial identification and susceptibility testing, we decided to include infections whose case definitions included criteria for culture confirmation to increase the surveillance system's sensitivity.

The feasibility of implementing surveillance consistently across the 6 hospitals was a major factor in deciding which infections to include in the surveillance system.

We developed HAI surveillance protocols for health care-associated BSIs, including central line-associated BSIs (CLABSI), and UTIs, including catheter-associated UTIs (CAUTI). Case definitions were adapted from NHSN's standardized definitions but streamlined to function within the contexts of these facilities.^11^

The 6 pilot hospitals had some experience conducting HAI surveillance, although approaches varied by hospital. They employed dedicated, full-time IPC staff, but these staff are often overburdened with other responsibilities, making it challenging to dedicate enough time to performing complex surveillance. Additionally, medical records in these hospitals often contain poor documentation of the dates that patients move between hospital units, making the implementation of surveillance rules that attribute an HAI to a particular unit challenging. To address these constraints, the modified BSI and UTI surveillance protocols only included infections in patients who had been in an ICU for at least 2 days, reducing surveillance complexity and the time required to identify a patient with an HAI.

The NHSN CLABSI case definition requires significant effort to rule out secondary BSIs, using criteria that include laboratory and imaging tests that are not readily available or used in Vietnamese hospitals. To reduce the burden required to identify and report a BSI and to address the inconsistent availability of diagnostic tests across surveillance hospitals, we modified the BSI case definition to classify infections as primary or secondary based purely on a patient's microbiological culture results.

We also attempted to make the process of identifying CLABSIs and CAUTIs more objective. Surveillance hospitals reported all BSIs and UTIs that met the case definition and were then automatically classified as device-associated or not device-associated by the surveillance system's online data reporting system based on standardized questions completed for each reported infection.

Finally, despite concerns about the burden of ventilator-associated pneumonia within Vietnamese hospitals relative to other HAIs,^12^ ventilator-associated pneumonia was not selected as a target for this HAI surveillance system due to the challenges of creating a simplified case definition that could be implemented consistently across surveillance hospitals.^13^

### Creating a Surveillance Implementation Strategy

**FIGURE 2:**
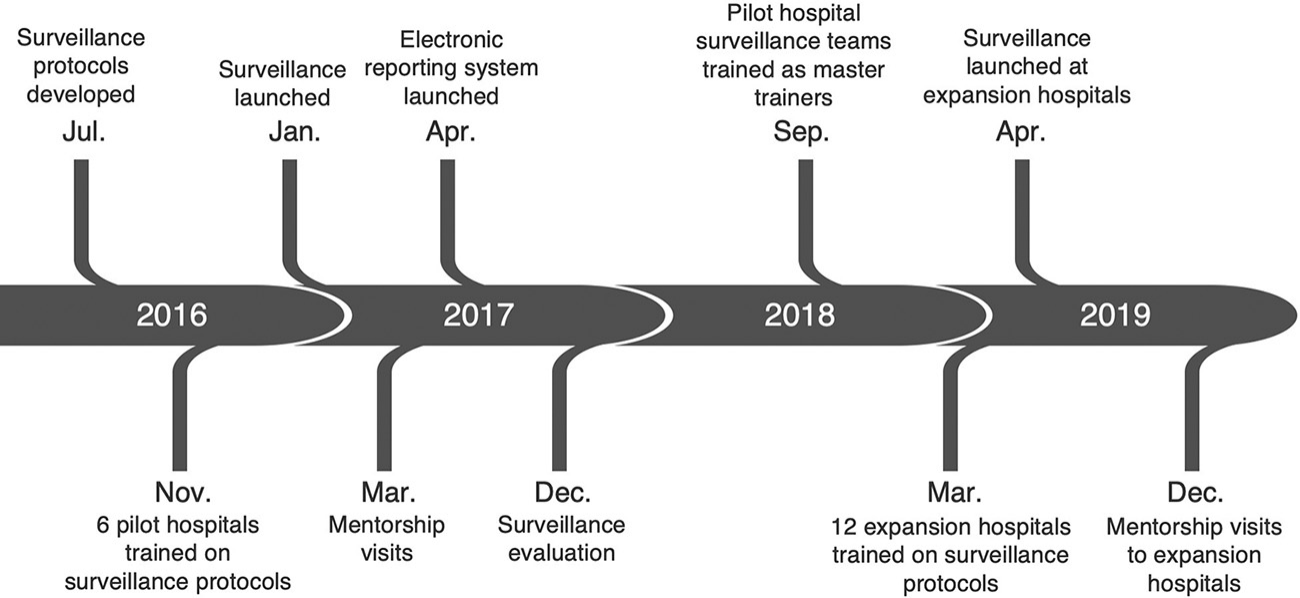
Timeline of Health Care-Associated Infection Surveillance Implementation Activities in 6 Pilot Hospitals, Vietnam

A methodical approach was used to implement BSI and UTI surveillance in 1–2 ICUs at each of the 6 pilot hospitals (Figure 2).

Hospital surveillance teams, including IPC, microbiology, and ICU staff, were trained on case definitions, case-finding procedures, data collection, and case reporting using a new electronic reporting system. By January 2017, participating ICUs had begun standardized surveillance for BSIs and UTIs. We conducted subsequent mentorship visits to the 6 hospitals within 3 months of starting surveillance to resolve surveillance teams' initial challenges before they became systemic.

The surveillance system was evaluated in December 2017 to assess hospitals' adherence to the surveillance protocols. The evaluation consisted of patient chart reviews to confirm the correct application of surveillance case definitions, interviews with hospital stakeholders to understand implementation successes and challenges, and ad hoc protocol training as needed. Evaluation results were used to address identified implementation gaps and inform future decisions regarding system expansion. In September 2018, leaders from the 6 pilot hospital surveillance teams were trained to become master trainers who can teach the surveillance protocols and conduct surveillance support visits as the surveillance system expands.

A gradual, stepwise approach for system expansion was prioritized to ensure strong implementation of surveillance protocols for any hospital joining the network and to preserve data quality. In the first phase of expansion, master trainers supported training, mentorship, and evaluation activities at 12 additional hospitals. By April 2019, a total of 18 hospitals were contributing data to the standardized HAI surveillance system with plans for continued phased expansion.

Expanding this network in a stepwise manner allowed for a focus on quality implementation.

### Linking HAI Surveillance and Prevention Activities

A primary objective of the standardized HAI surveillance system for BSIs and UTIs was to support BSI and UTI prevention efforts at the 6 hospitals. All 6 hospitals had some baseline IPC capacity, which includes established IPC teams and committees, dedicated IPC staff, and microbiology laboratory support.

As part of surveillance implementation, hospital IPC teams are learning to use the electronic reporting system to look for errors in surveillance data quality to understand how and when trends can inform prevention targets. Following a systematic assessment of the knowledge of CLABSI and CAUTI “bundles of care,” the 6 hospital IPC teams developed and implemented focused IPC improvement projects using a quality improvement approach. For example, 1 hospital implementing a CLABSI prevention project closely followed their monthly HAI rates and noted that CLABSI rates had more than doubled in 1 of their pediatric ICUs over a 2-month period. An investigation identified the use of multidose saline bottles and inadequate environmental cleaning as potential factors. After mitigating these risks, CLABSI rates returned to baseline. This demonstrates how continued use of HAI surveillance data can inform the progress and direction of targeted prevention activities.

## IMPLEMENTATION CHALLENGES

We encountered several challenges during the pilot implementation of the standardized HAI surveillance system in 6 hospitals.

### Competing Priorities for Hospital-Based IPC Leaders

IPC leaders at these pilot hospitals have been crucial to establishing and expanding the system, but they have needed to balance these activities with their responsibilities coordinating all IPC activities at their respective hospitals, advising VAMS on national IPC policies and guidelines, and supporting smaller local hospitals with IPC training and mentorship. VAMS and hospital leadership have allowed hospital IPC leaders to devote focused time to early implementation and expansion efforts. IPC leaders' competing priorities also underscore the need to create surveillance protocols and training materials that reflect local human resource limitations and the need to develop new experts who can be leveraged for continued sustainable expansion efforts, ensuring that workload for training and mentoring is distributed to more individuals as new surveillance sites are added to the network.

### Integration of New Surveillance Into Existing Workflows

During early implementation, hospital surveillance teams faced challenges integrating new surveillance processes into their existing workflows. For instance, the 1-year evaluation of the surveillance system demonstrated that positive blood and urine culture results were not always reliably communicated from the microbiology laboratory to the surveillance team at several pilot hospitals, thereby reducing the sensitivity of the surveillance system to detect all BSIs and UTIs that met the case definition. As a result, protocols requiring microbiology laboratories to review blood and urine culture data each month to ensure that complete results are provided to surveillance teams were developed and implemented in the 6 pilot hospitals and have been added to trainings for new hospitals joining the surveillance network.

### Inconsistent Application of Surveillance Between Sites

The surveillance evaluation also revealed that some hospital surveillance teams had encountered scenarios for which the protocols and training had not provided sufficient guidance, resulting in inconsistent application of surveillance protocols across sites, such as different interpretations of criteria for contaminated urine cultures that did not meet the UTI case definition. Ad hoc site visits to receive feedback from hospital stakeholders, review case finding challenges, and observe surveillance data flow processes proved to be important opportunities to identify and understand the causes of this inconsistent application. These visits allowed VAMS, CDC, and PATH to provide on-the-spot training to reinforce protocol fundamentals and informed the development and dissemination of additional tools and guidance materials to all hospitals to promote consistent protocol implementation. Questions that were asked during site visits and implementation gaps that we identified were used to develop a frequently asked questions document that was shared with all 6 hospitals to improve consistency. We saw consistent implementation of surveillance across hospitals as a key priority. Developing a system of intensive on-site mentorship at participating hospitals was critical in maintaining this consistency and ensuring data quality. As the system expands, we are taking a phased, stepwise approach to ensure that adequate mentorship and monitoring and evaluation can be conducted at newly enrolled hospitals.

Developing a system of intensive on-site mentorship at participating hospitals was critical in maintaining consistent implementation of surveillance and ensuring data quality.

## LESSONS LEARNED

We implemented a standardized HAI surveillance system for BSIs and UTIs that will help quantify the burden of disease and allow for targeted HAI prevention interventions in Vietnam. Over the course of 2 years, Vietnam rolled out standardized BSI and UTI surveillance in select ICUs at 6 hospitals, establishing an important foundation for a national HAI surveillance system.

Maintaining stakeholder investment in establishing the foundation for Vietnam's national HAI surveillance system will be key to its growth and longevity. We have received early positive feedback on the standardized system's simplicity, with several hospitals having replaced previous surveillance methods with that of the standardized system in ICUs outside of those we are currently engaging. By addressing a local need, aligning priorities with national and hospital stakeholders, and tapping into the technical expertise of local and international stakeholders, we are building the foundation for sustainability.

Having clear roles and responsibilities was important. As champions in the HAI surveillance network, VAMS and the 6 hospital IPC teams have played leadership roles in training additional hospitals as the surveillance system gradually expands. Technical partners have been building local capacity at both the national and hospital levels to maintain, monitor, and eventually expand the surveillance system.

One critical aspect was the development of case definitions that could be applied consistently across surveillance hospitals based on the local context to ensure the quality of data generated by the surveillance system. HAI surveillance protocols provided by U.S. CDC and ECDC include case definitions that use imaging and laboratory criteria commonly found in highly resourced facilities in highly resourced countries. Their use in lower-resource settings, where these tests are not available or not used, will produce inaccurate data. Modifications made to the HAI surveillance protocols in Vietnam to address the local context, particularly human resource constraints, have led to more consistent implementation across hospitals.

The initial focused implementation of the HAI surveillance system in a limited number of ICUs at each hospital made it more likely to ensure long-term sustainability of surveillance. Integrating new surveillance processes into a facility's existing workflow requires time and substantial effort, which was more manageable with fewer participating ICUs. Furthermore, implementing surveillance on a small scale allowed us to effectively address challenges hospital surveillance teams encountered through focused, intensive mentorship. Through this methodical approach, sustained incremental successes in starting standardized HAI surveillance at the 6 hospitals were demonstrated.

Demonstrating the essential link of HAI surveillance to HAI prevention programs was also an essential component. We worked with surveillance hospitals to teach them the value of HAI surveillance data in the context of their HAI prevention programs and to implement focused monitoring of select HAI prevention efforts. Building concurrent capacity for outcome and process surveillance systems ensures that HAI surveillance is a critical component of a larger system for HAI prevention.

## CONCLUSION

VAMS continues to support hospitals conducting standardized HAI surveillance. By acquiring and maintaining stakeholder buy-in and investing resources into the 6 pilot hospitals, we overcame 2 threats to successful early system implementation. In collaboration with technical partners, VAMS empowered and equipped IPC experts from the 6 pilot hospitals to function as support providers for additional hospitals that join the surveillance network. IPC leaders from the 6 pilot hospitals were trained as master trainers and now support newly joined sites by delivering surveillance protocol and master trainer trainings, answering questions, and conducting periodic in-person visits to these new sites to assist with surveillance implementation. CDC and PATH continue to function as technical advisors for the surveillance system. Thus, with the active participation of IPC staff from the 6 initial hospitals, this standardized HAI surveillance system for BSIs and UTIs has been expanded to 12 additional hospitals, with the ultimate goal of developing a national network. To date, 18 hospitals are reporting BSI and UTI surveillance data with reduced dependency on external support. Together, VAMS and partners are helping Vietnam fulfill its commitment to safe health care for all patients.

## Notes

**Peer Reviewed**

**First published online:** June 1, 2022.
